# Formin and capping protein together embrace the actin filament in a ménage à trois

**DOI:** 10.1038/ncomms9730

**Published:** 2015-11-13

**Authors:** Shashank Shekhar, Mikael Kerleau, Sonja Kühn, Julien Pernier, Guillaume Romet-Lemonne, Antoine Jégou, Marie-France Carlier

**Affiliations:** 1 Cytoskeleton Dynamics and Cell Motility, Department of Biochemistry, Biophysics and Structural Biology, I2BC, CNRS, 91198 Gif-sur-Yvette, France

## Abstract

Proteins targeting actin filament barbed ends play a pivotal role in motile processes. While formins enhance filament assembly, capping protein (CP) blocks polymerization. On their own, they both bind barbed ends with high affinity and very slow dissociation. Their barbed-end binding is thought to be mutually exclusive. CP has recently been shown to be present in filopodia and controls their morphology and dynamics. Here we explore how CP and formins may functionally coregulate filament barbed-end assembly. We show, using kinetic analysis of individual filaments by microfluidics-assisted fluorescence microscopy, that CP and mDia1 formin are able to simultaneously bind barbed ends. This is further confirmed using single-molecule imaging. Their mutually weakened binding enables rapid displacement of one by the other. We show that formin FMNL2 behaves similarly, thus suggesting that this is a general property of formins. Implications in filopodia regulation and barbed-end structural regulation are discussed.

## Abstract (web summary)

Formins promote actin filament polymerization and capping protein blocks polymerization; both proteins are thought to exclude each other from barbed ends. Here the authors show that both proteins can simultaneously bind barbed ends in a ternary complex while enhancing each other's dissociation from the barbed end.

## Graphical abstract

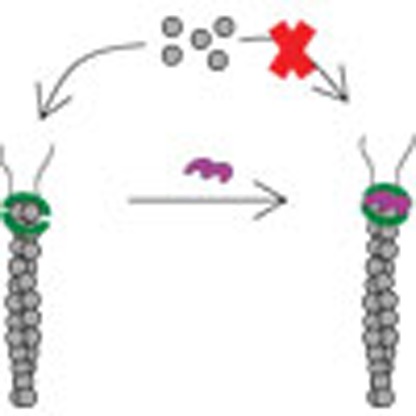

Polarized assembly of actin filaments is pivotal in cell motility and is orchestrated by proteins that target filament barbed ends^1^. Formins bind barbed ends and accelerate filament elongation from profilin-actin (PA) in a processive fashion^2^,^3^,^4^. In contrast, capping protein (CP) blocks filament growth upon its binding to barbed ends. Both proteins together regulate cell migration^5^,^6^,^7^,^8^. Formins and CP are thought to bind the barbed end of a filament in a mutually exclusive fashion, that is, each protein prevents the other from binding the barbed end of the actin filament^9^,^10^,^11^,^12^. Bulk solution assembly assays^9^ demonstrated that formin had to remain bound to the barbed end during at least 10 cycles of actin assembly to account for the protection against capping. However, it was also noted, but not explained, that the barbed-end protection by formin depended on the relative amounts of CP and formin. In addition, Romero *et al.*^3^ showed that Gelsolin arrested propulsion of formin-coated beads in a concentration-dependent fashion and that the affinity of CapG for barbed ends reached a lower limit at saturating levels of formin, at variance with the unlimited decrease expected for mutually exclusive binding. These data suggested that the two proteins simultaneously bind barbed ends^3^. However, the underlying molecular mechanism and kinetic details have so far remained elusive.

Formin and CP individually dissociate extremely slowly from barbed ends^3^,^13^, which raises intriguing questions regarding the regulation of filament length in formin-based motile processes. Given the dwell times of formins at the barbed ends (approximately tens of minutes) obtained from *in vitro* experiments^14^, formin-based cellular processes should reach lengths of several tens of microns before formin detachment. Yet in filopodia, bundles of actin filaments assembled at 30–500 nm s^−1^ (ref. 15) by processive polymerases such as formin mDia2 or Ena/VASP proteins^16^,^17^,^18^,^19^,^20^, reach lengths of a few microns. Similar to Bud14, which displaces formin Bnr1 (ref. 21) in yeast, a mechanism to reduce formin dwell times at the barbed end in eukaryotes must exist. In contrast to previously held views^22^, CP has recently been reported to be present in filopodia^8^. This finding prompted us to take a fresh look at the presumed competition between CP and formin at filament barbed ends.

Here we show that CP and formin mDia1 can together bind to actin filament barbed ends in a ternary complex in which CP acts as an uncompetitive inhibitor of formin. The dynamics of formin and CP in complex with the barbed end have been analysed using microfluidics-assisted single-filament assay^23^,^24^. The ternary complex has been further visualized using single-molecule fluorescence imaging. The mutually weakened binding of CP and formin to barbed ends promotes rapid displacement of formin from barbed ends by CP and uncapping of CP by formin. We propose CP and formins as potential novel cross-regulators of each other in cellular organelles like lamellipodia, cytokinetic ring and filopodia, where the two proteins are known to play a vital role.

## Results

### Two set-ups reveal interplay of formin and CP at barbed ends

We have analysed the interplay of mDia1 FH1–FH2–DAD and CP at barbed ends of individual actin filaments using a home-built microflow-based set-up^25^. In rest of the text the notation ‘mDia1' refers to FH1–FH2–DAD domains of formin mDia1, unless otherwise specifically mentioned. Similarly FMNL2 refers to the FH1–FH2-WH2-DAD domains of formins FMNL2.

Filaments were either initiated from immobilized spectrin–actin seeds, exposing their distal barbed ends to PA, formin or CP at defined concentrations (set-up #1), or from surface-anchored formins, initiating insertional processive barbed-end assembly from PA and CP (set-up #2). The two set-ups are complementary to each other. The first set-up allows us to monitor the changes in barbed-end elongation rate caused by the association of CP (C) or formin to a free barbed end (B), and by association of one of the two proteins to a barbed end bound to the other one. We can further monitor the evolution of barbed-end dynamics following removal of formin and CP. The second set-up, where the formin is anchored (BF), allows us to observe the change in elongation rate due to the binding of CP to a formin-bound barbed end and quantify its evolution into a CP-bound or a formin-bound barbed end in absence of the two proteins. Three input flows are used, each consisting of a specific biochemical composition, for example, PA (flow 1), formin (flow 2) and CP with PA (flow 3). The biochemical conditions can be changed in less than a second^24^. Analysis of the kymographs of large number of filaments at varying CP and formin concentrations allow studying molecular mechanisms with high accuracy. In other words, the method enables single-molecule kinetics without the need of labelling individual protein molecules.

### CP binds to formin-bound barbed ends in a ternary complex

**Figure 1:**
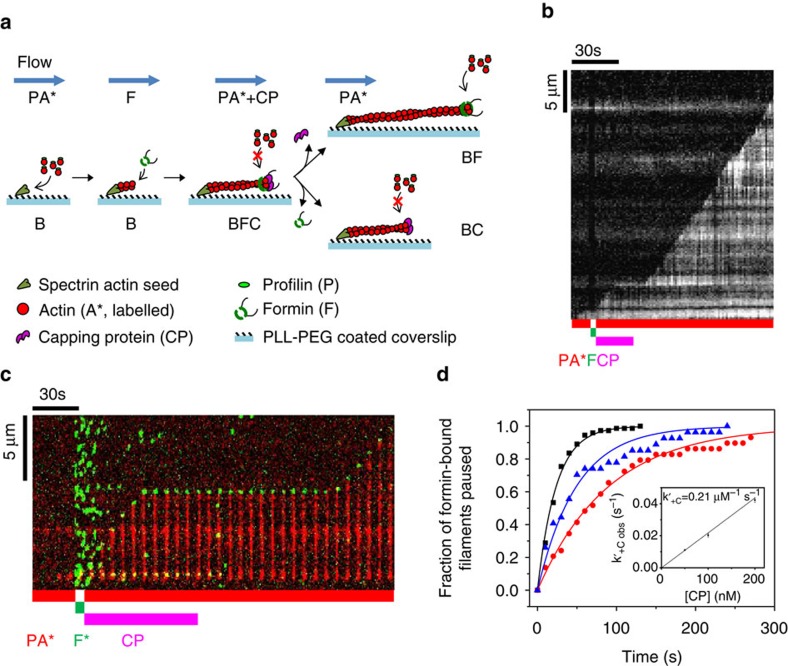
CP associates to mDia1 formin-bound barbed ends in a capped ternary BFC complex that releases either formin or CP. (**a**) Schematic representation of experimental set-up #1. Barbed-end growth is initiated from spectrin–actin seeds bound on the coverslip surface. The filaments are then sequentially exposed to flows containing PA, formin, CP as indicated. (**b**) Kymograph of a filament showing evidence for the pathway BF+C→BFC→BF+C with unlabelled formin and CP. In the kymograph, an actin filament exposed to 20 nM formin (F) for 10 s exhibits fast elongation in the presence of PA. On exposure to 100 nM CP (CP+PA) for 20 s, filament growth is arrested. On exposure to PA, CP dissociates leaving behind fast-elongating BF. (**c**) Similar kymograph as in **b** but with fluorescently labelled formin F*. Filaments growing in PA (red) were exposed to 20 nM labelled formin F* (green) for 10 s after which filaments were re-exposed to PA. The higher green background appears to stay for slightly longer (∼30 s) even after formin is removed from the flow due to non-specific interaction of formin with the surface. Formin can be seen bound to the barbed end in the paused state in the BF*C complex as a result of exposure to PA+CP. Fast processive elongation resumes when CP falls off (BF*C→BF*+C) and the same formin continues to processively track the barbed end (Supplementary Movie 1). (**d**) Fraction of rapidly elongating BF filaments converting to arrested BFC state on binding CP at the following concentrations (nM): 200 nM (black symbols, *n*=36 filaments), 100 nM (blue symbols, *n*=27 filaments) and 50 nM (red symbols, *n*=29 filaments). Symbols represent the experimental data and the solid lines are the exponential fits providing *k*′_obs_ values. Inset: plot of *k*′_obs+C_ versus [CP] giving *k*′_+C_=0.21±0.01 μM^−1^·s^−1^ (see Supplementary Fig. 3 for histograms. Error bars: s.e.m.).

Using set-up #1 (Fig. 1a), filaments were elongated from glass-anchored spectrin–actin seeds in the presence of PA, the freely floating end being the barbed end (B). Exposure to a flow containing mDia1 (F) alone led to the formation of a formin-bound barbed end (BF). On subsequent exposure to PA alone, fast processive assembly was observed. Formin-bound filaments (BF) were then exposed to a flow of CP and PA in the absence of mDia1. The fast processive growth of filaments was arrested by CP, surprisingly at a much faster rate than if binding of CP was rate limited by the very slow dissociation of formin, which would be expected for a mutually exclusive binding scheme (Fig. 1b,c). The first-order rate constant at which growth was arrested in contrast increased linearly with CP concentration, consistent with a simple bimolecular reaction (Fig. 1d), indicating that CP binds formin mDia1-bound barbed ends (BF) and arrests their growth in a Barbed end-Formin-CP complex (BFC) state, as described by the following scheme: 
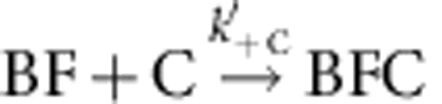
. CP effectively caps the barbed end in the BFC state. Note that 100% of the BF filaments (total *n*=80–100 filaments) was converted into the arrested BFC complex after exposure to CP. The BFC filaments, when exposed to a flow containing only PA (no free CP and no free formin), partitioned into two populations. In all, 30% of BFC filaments rapidly resumed fast processive assembly (indicative of formin-based elongation), the remaining ones slowly resumed typical free barbed-end growth from PA. We conclude that in the capped BFC complex both CP and mDia1 interact with the barbed end via the terminal actin subunits, without any direct interaction with each other (Supplementary Fig. 1). The BFC complex either undergoes dissociation of CP leading to formin-mediated fast processive assembly (
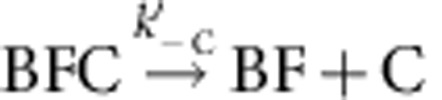
), for which a single exponential was recorded (*k*′_−C_=1.8±0.07 × 10^−3^ s^−1^; Table 1), or dissociation of mDia1 restoring the CP-capped state (BC) that then slowly releases CP, leading to free barbed ends (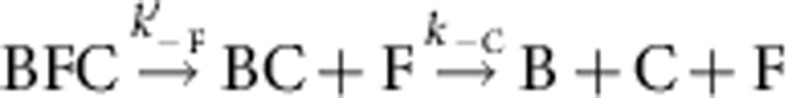). Figure 1b shows a kymograph of a filament paused in a BFC state that resumes elongation in the BF state on dissociation of CP. Note that only the outcome BFC→BF+C can be readily observed using set-up #1, as BC produced by BFC→BC+F is undistinguishable from BFC. Nevertheless, the simplest interpretation of the fact that only up to 30% of BFC dissociates into BF is that 70% of BFC follows the pathway BFC→BC+F, as observed later using set-up #2.

Further evidence for arrested barbed-end growth in the ternary BFC complex was obtained from single-molecule microscopy using fluorescently labelled mDia1 (denoted by F*). Filaments nucleated from spectrin–actin seeds were transiently exposed to fluorescently labelled mDia1 (B+F*→BF*). When the BF* filaments were exposed to a flow containing CP and PA, barbed-end growth was arrested while the fluorescent formin stayed bound to the barbed end, thus confirming that CP and mDia1 were simultaneously bound to the barbed end. On CP removal from the flow, the BF*C filament exposed to PA switched back to rapid elongation. This established that the same formin molecule stayed bound to the barbed end before, during and after the pause (Fig. 1c and Supplementary Movie 1). Labelling of mDia1 did not affect the rate of processive elongation (44.2±6.3 and 45.3±7.3 subunits per second at 1 μM actin, 4 μM profilin, for unlabelled and labelled formin, respectively; *n*=50 filaments). Tests to check step bleaching confirmed that labelled formins were indeed single molecules and not aggregates (Supplementary Fig. 2). While single-molecule imaging provides qualitative demonstration of the ternary barbed-end formin–CP complex, the low labelling fraction of formins (∼5%) prevent quantitative measurements. The kinetic analysis of CP binding to BF was therefore performed using unlabelled actin-binding proteins.

The value of the rate constant for CP association to formin-bound barbed ends was *k*′_+C_=0.21±0.01 μM^−1^·s^−1^, as derived from the linear increase in the pseudo first-order rate constant measured for the reaction BF+C→BFC versus CP concentration (Fig. 1d, Supplementary Fig. 3 and Table 1). The rate constants for association of formin and CP to free barbed ends were similarly derived (Supplementary Figs 4a and 5a, respectively, and Table 1).

### Either CP or formin dissociate rapidly from the BFC complex

**Figure 2:**
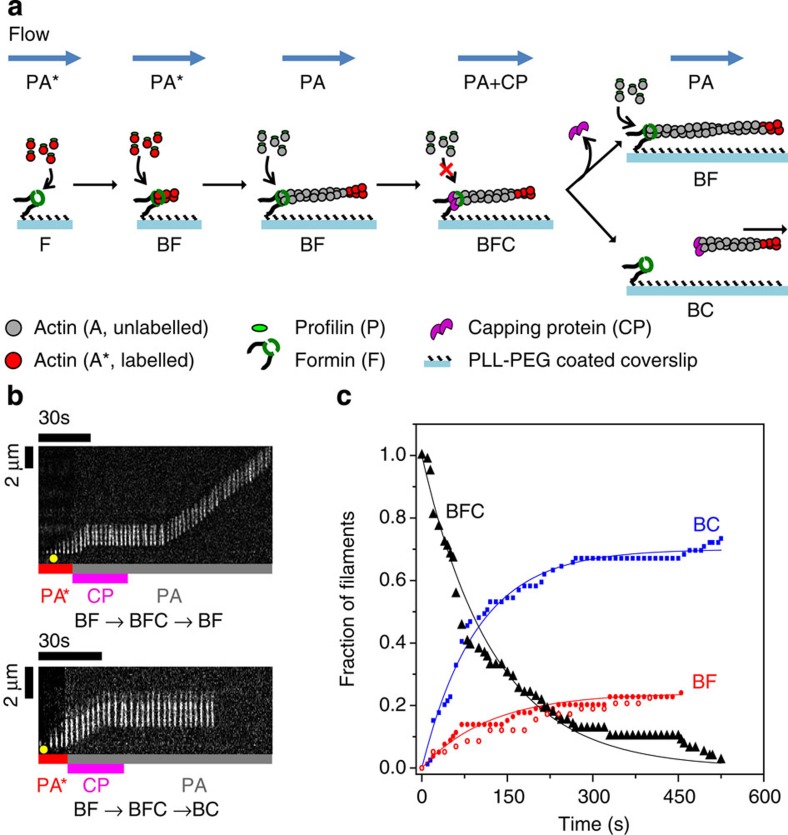
The BFC state splits into CP-capped (BC) and formin mDia1-bound (BF) states. (**a**) Schematic representation of the experimental set-up #2. Fluorescent actin filaments are initiated from formins anchored on the coverslip. The filaments are then sequentially exposed to flows containing PA (non-fluorescent) and CP as indicated. (**b**) Kymographs of two formin-anchored (indicated by the yellow dot) filaments switching from the rapidly elongating state (BF) to the pausing state (BFC) on binding CP, followed by the transition to either BF (top) or BC (bottom). Rapidly elongating filaments were initiated by exposing formins to the fluorescent actin and profilin. Filaments were then exposed to a solution containing PA with non-fluorescent actin and 100 nM CP for 30 s. On removal of CP from the flow and introduction of non-fluorescent PA, filaments either resume fast elongation (BFC→BF) or detach (BFC→BC). Elongation by formin in non-fluorescent actin gives the appearance of ‘fluorescent segment' moving further away. (**c**) Fraction of BFC filaments (black symbols; *n*=76 filaments) undergoing dissociation into either BC (filaments released in the flow, blue squares) or BF (filaments resuming fast growth, red circles). For comparison, the time course of BF produced from BFC in set-up #1 (Fig. 1) is plotted (open red circles). The solid lines are the exponential fits. The data were fitted with an exponential process (continuous line) consistent with a rate constant *k*_obs_=*k*′_−F_+*k*′_−C_.

Formation of the ternary BFC complex was tested using set-up #2 (Fig. 2a), which may mimic the condition in which the formins are activated and immobilized at a membrane. In contrast with set-up #1, set-up #2 allows us to clearly distinguish the capped BFC state (attached filament) from the capped BC state (detached filament, lost in the flow) and the BF state (resumption of fast elongation).

The filaments were first nucleated in fluorescent PA, and then non-fluorescent PA+CP was introduced. Since the monomer addition takes place in an insertional fashion between the formin and the pre-existing filament, the fluorescent segment appears to move away from the immobilized formin as non-fluorescent monomers get added at the barbed end by anchored formins^25^. Processive assembly of 100% of formin-anchored filaments (*n*=76 filaments) was arrested by exposure to CP, confirming the conversion of formin-bound barbed ends (BF) into the capped BFC state. The rate constant for CP binding to BF was derived from experiments performed at various CP concentrations. The measured value *k*′_+C_=0.08±0.0012 μM^−1^·s^−1^ is slightly lower than the one derived from set-up #1 (0.21±0.01 μM^−1^·s^−1^). The difference might result from the anchoring of the formin as well as the use of labelled actin in set-up #1 and unlabelled actin in set-up #2. The rate constant for association of CP to formin-bound barbed ends (*k*′_+C_) was two orders of magnitude lower than for CP binding to free barbed ends (*k*_+C_; Table 1). *k*′_+C_ was also found to be identical at two different PA concentrations (Supplementary Fig. 6). On exposure of arrested BFC filaments to PA, the BFC filaments partitioned into detached BC filaments and rapidly growing BF filaments (Fig. 2b). The low flow rate prevented filament detachment due to viscous drag. In addition, formin-anchored filaments spontaneously detach from formins at a rate which is orders of magnitude lower (Table 1). It was further confirmed that filament detachment was due to the dissociation of the capped barbed end from the formin, and not due to the dissociation of formin from the surface (mediated by the strong neutravidin–biotin link). This point was verified by renucleating new filaments from the anchored formins from which filaments had detached following BFC formation. These formins were able to re-nucleate new filaments (Supplementary Fig. 7 and Supplementary Movie 2). The kinetics of dissociation of BFC into BC and BF was exponential with rate constant *k*_obs_=*k*′_−F_+*k*′_−C_ (Fig. 2c). The final fractions of filaments in BF and BC states are *k*′_−C_/(*k*′_−C_+*k*′_−F_) and *k*′_−F_/(*k*′_−C_+*k′*_−F_), respectively (see equations 1, 2, 3 in Methods section). Kinetic analysis of the fraction of filaments in BFC, BF and BC states provided values of 6.34±0.09 × 10^−3^ s^−1^ and 2.02±0.04 × 10^−3^ s^−1^ for *k*′_−F_ and *k*′_−C_, respectively. Superimposable curves for BFC→BF were obtained with both set-ups (Fig. 2c, open and closed red symbols). Although dissociation of CP from BFC is not directly measured, but inferred from the formation of rapidly elongating BF, the truly monoexponential formation of rapidly elongating BF state rules out a kinetic scheme in which rapid dissociation of CP would lead to an intermediate inactive (non-growing) BF′ state of the barbed end, followed by the actually monitored slower structural re-arrangement leading to active BF. Note also that following exposure to PA only, the evolution of 100% BFC is well-described by the partition between two functionally characterized states BF and BC. No evidence was detected for a second inactive BF′ state occurring in a side reaction. Thus, dissociation of CP from BFC leaves formin readily active at the barbed end.

In conclusion, formin dissociates 78-fold faster from CP-bound barbed ends than from free barbed ends (*k*_−F_=8.1±0.35 × 10^−5^ s^−1^, Supplementary Fig. 4b). The monoexponential time courses of BF appearance are reasonably superimposable in set-up #1 and set-up #2. Two important conclusions are arrived at. First, anchoring of formin to the glass surface does not affect BFC dynamics (Fig. 2c). Second, the same partitioning of BFC into BC and BF is monitored with both set-ups, hence the rate constants *k*′_−C_ and *k*′_−F_ for CP and formin dissociation from BFC can be derived from kinetic analysis of data from set-up #1 as well as from set-up #2 using equations (1, 2, 3) (see Methods section), even though no direct measurement of *k*′_−F_ is obtained in set-up #1.

The large decrease in affinity of CP for barbed ends due to bound formin is mainly caused by a 60-fold decrease in association rate (only 9–10-fold increase in dissociation rate in both set-ups). The apparently very slow association rate constant of CP to BF is suggestive of a two-step binding, rapid equilibrium followed by a slower structural re-arrangement enhancing the strength of binding.

### Formin binds to CP-capped filaments to form a BCF complex

**Figure 3:**
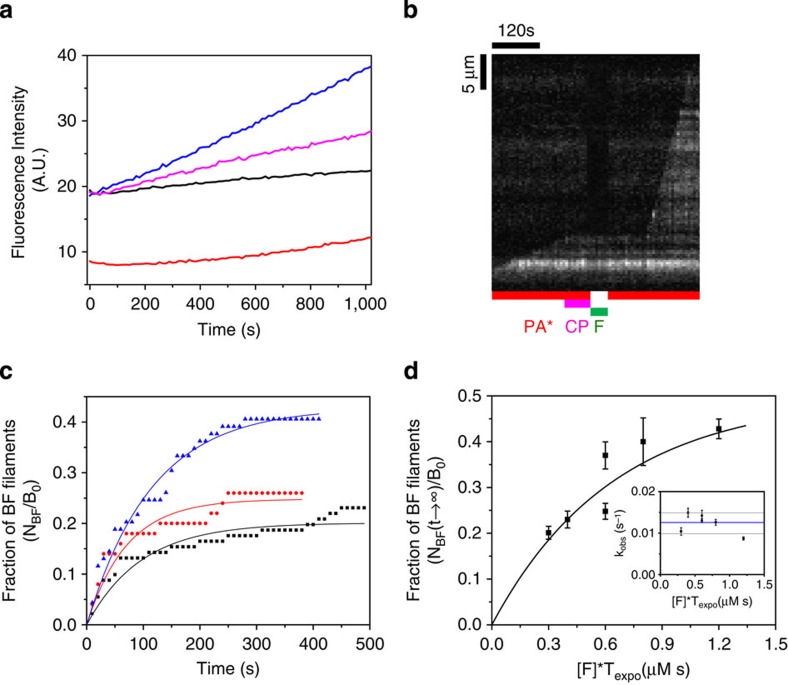
Formin mDia1 binds to CP-capped filaments and rapidly uncaps via a transient BCF state. (**a**) Pyrene actin polymerization assay demonstrates uncapping of capped barbed ends (BC), by formin. Capped filaments (5 μM F-actin, 2% pyrenyl labelled and 5 nM CP) were diluted 50-fold in F-buffer containing 2 μM G-actin (2% pyrenyl labelled) and 6 μM profilin and the following additions: none (black), 2 nM formin (blue) and 4 μM CIN85 (magenta). Red curve is a filament nucleation control (2 μM actin, 6 μM profilin and 2 nM formin) in the absence of CP-capped filaments. Note that a small percentage of non-capped filaments are responsible for the non-zero initial rate in the black (free barbed ends) and blue (formin-bound barbed ends) curves. Dead time is about 20 s. (**b**) Kymograph of a capped filament undergoing uncapping and fast processive growth on exposure to formin mDia1. Filament was elongated from anchored spectrin–actin seeds in the presence of PA, then exposed to 20 nM CP and PA for 1 min (B+C→BC). The capped filament is later exposed to 40 nM formin in the absence of PA for 40 s (BC+F→BCF). Once formin was removed from the flow and PA was introduced, fast elongation was observed (BCF→BF+C). (**c**) Fraction of filaments (in experiment described in **b**) that resume rapid elongation (BF state) during exposure to PA only, versus time, from an initial population of capped filaments exposed to 10 nM (black, *n*=91 filaments), 20 nM (red, *n*=50 filaments) and 40 nM (blue, *n*=69 filaments) formin for 30 s. Symbols represent the experimental data and the solid lines are the exponential fits. Only three representative CDFs are shown here for the ease of reading, see Supplementary Fig. 9 for details. (**d**) The maximum fraction of BF filaments (plateau values of curves such as shown in **c**) as a function of formin concentration [F] times the exposure duration (*T*_expo_). The solid line is an exponential fit corresponding to equation (3). Inset: *k*_obs_=*k*′_−C_+*k*′_−F_ is independent of the experimental condition. Horizontal lines represent the average (blue) plus or minus the s.d. (grey). Error bars: s.e.m.

We then investigated if mDia1 could also associate to CP-capped barbed ends to form a ternary BCF complex and if BCF is biochemically equivalent to BFC. This was first tested in bulk solution assays by diluting (50-fold) Pyrenyl-labelled CP-capped filaments in F-buffer containing PA (Fig. 3a). The dilution decreased the concentration of free CP to <100 pM, thus making the re-binding of CP to barbed ends following its dissociation negligible. Expectedly, capped filaments failed to grow significantly in the presence of 2 μM PA. However, when mDia1 was added to the mix of capped filaments and PA, processive actin polymerization occured and caused an acceleration, in conditions where mDia1 nucleation is negligible (Fig. 3a). The acceleration matches the time course of CP displacement by formin. No such acceleration was observed when rapid uncapping was elicited by CIN85, a conventional uncapper of the CapZIP family^26^,^27^, thus this control curve bends downward in a conventional first-order growth process (Fig. 3a). This result provides qualitative indication that mDia1 binds capped barbed ends and uncaps CP, promoting fast growth, before CP has time to dissociate spontaneously (Supplementary Fig. 5b).

When dilution-induced disassembly of the same CP-capped filaments was tested in F-buffer containing profilin only, no uncapping by mDia1 was observed. In contrast, CIN85 did promote uncapping and profilin-enhanced barbed-end disassembly (Supplementary Fig. 8).

Single-filament assay was then used to quantify formin-based uncapping. The formation and outcome of the BCF state was performed and analysed as follows. A population of B_0_ filaments were grown from immobilized spectrin–actin seeds (set-up #1) and 100% capped by CP. These filaments were exposed to formin for a given duration *T*_expo_. Both the concentration of formin [F] and incubation period *T*_expo_ were varied, leading to various resulting amounts of BCF via the reaction 
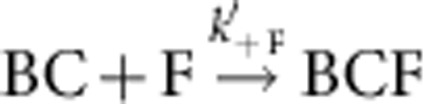
. Following the incubation period *T*_expo_ of BC with F, PA was flown in at time zero, allowing a fraction of the preformed BCF filaments to convert into fast-growing BF state. A kymograph of a BCF filament evolving into BF is shown in Fig. 3b. Note that following exposure to F, this filament remains in the arrested BCF state for several seconds when exposed to PA alone, before fast processive assembly starts. We assume that during the period *T*_expo_, the reaction BC→B+C, which is very slow, can be neglected, as well as the reactions BCF→BC+F and BCF→BF+C (which is verified a *posteriori*, see below). The fraction of the initial capped B_0_ filaments that converted to BCF state was thus equal to 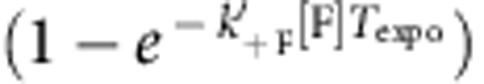. The conversion of BCF filaments into BF is described by equation (2) (see Methods section), providing a value of (*k*′_−C_+*k*′_−F_) identical to the one found in Fig. 2.

At sufficiently long time of exposure to PA (*t*→∞), the fraction of B_0_ filaments in the BF state plateaued at a value that depends on the concentration of formin [F] and exposure time *T*_expo_ (Fig. 3c and Supplementary Fig. 9), as described by the following equation:


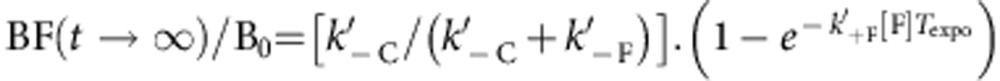


The values of *k*′_+F_ and *k*′_−C_ were derived from the fit of the data using this equation (Fig. 3d). We have thereby determined that *k*′_+F_=1.6±0.5 μM^−1^·s^−1^. Values found for the dissociation rate constants of CP and formin from the BCF state were *k*′_−C_=6.2±1.1 × 10^−3^ s^−1^, and *k*′_−F_=6.2±1.1 × 10^−3^ s^−1^ (Table 1), in satisfactory agreement with values found for dissociation of CP and formin from the BFC complex.

In this analysis, we have neglected reactions BC→B+C, BCF→BF+C and BCF→BC+F during the exposure of BC filaments to F. This simplification is justified by <5% detectable BF filaments at time *t*=10 s following exposure to PA (Fig. 3c). This result is in agreement with bulk solution measurements indicating that the dissociation of CP by formin (BCF→BF+C) requires PA (Supplementary Fig. 8).

In conclusion, CP and formin can together bind barbed ends irrespective of their order of binding. The affinities of both CP and formin for the barbed end are reduced by three orders of magnitude in the ternary BFC/BCF complex, which expresses the uncompetitive inhibition of formin activity by CP. The kinetic parameters of the ternary complex obtained by binding F to BC or C to BF are comparable (Table 1). The energetic costs for formation of the ternary complex via binding of F to BC or C to BF were calculated using the average values of equilibrium dissociation constants derived from rate parameters (Table 1). We find that *K*_C_·*K*′_F_∼*K*_F_·*K*′_C_, expressing detailed balance.

### FMNL2 formin also binds CP-capped filaments

Is formation of a BFC/BCF ternary complex an exclusive feature of mDia1 or does it represent a conserved, general behaviour of formins? FMNL2 is another Rho GTPase-activated, mammalian formin that localizes, as CP, in lamellipodia and filopodia^28^. FMNL2 and CP were tested for their simultaneous binding to barbed ends. FMNL2 by itself poorly nucleates actin filaments and binds barbed ends with lower affinity than mDia1, yet it processively elongates barbed ends in the presence of PA with a twofold increase in filament growth rate^28^.

**Figure 4:**
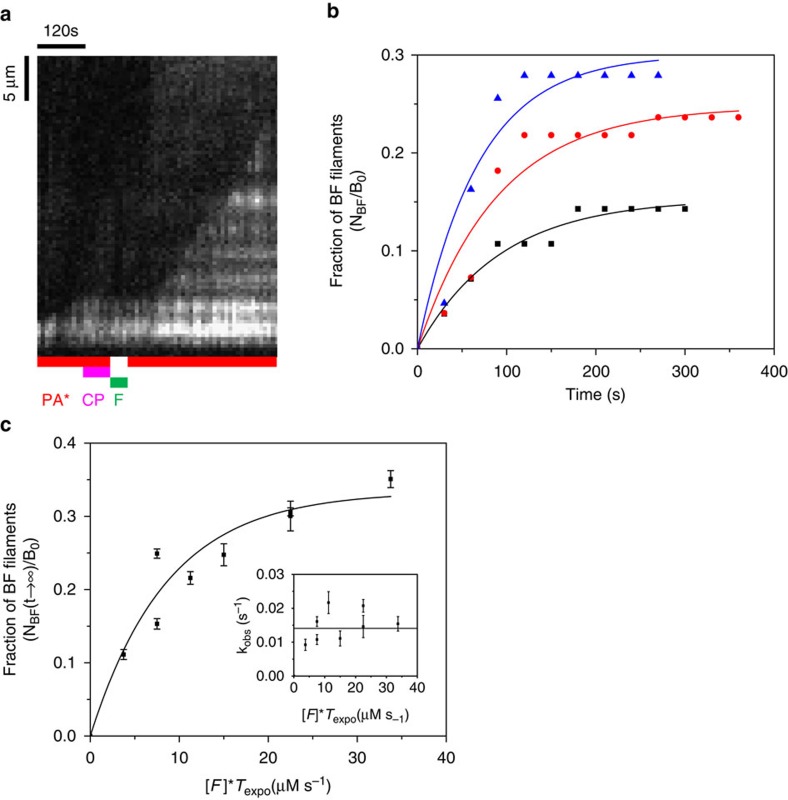
Formin FMNL2 also binds to CP-capped filaments and uncaps via a transient BCF state. (**a**) Kymograph of a capped filament undergoing uncapping and fast processive growth on exposure to formin FMNL2. Filaments elongating from spectrin–actin seeds (set-up #1, Fig. 1a) were first exposed to 20 nM CP and PA for a couple of minutes (B+C→BC). Paused filaments were then exposed to 250 nM FMNL2 for 30 s (BC+F→BCF). Once formin was removed from the flow and PA was introduced, fast elongation was observed (BCF→BF+C). Note that, as expected, the FMNL2 elongation rate seen here is much slower compared with that of formin mDia1 as seen in Fig. 3b. (**b**) Fraction of CP-capped paused filaments that resume rapid elongation (BF state) during exposure to PA only, versus time, from an initial population of capped filaments exposed to 250 nM (black, *n*=56 filaments), 500 nM (red, *n*=55 filaments) and 750 nM (blue, *n*=43 filaments) FMNL2 for 30 s. Symbols represent the experimental data and the solid lines are the exponential fits. Only three representative CDFs are shown here for the ease of reading, see Supplementary Fig. 10 for details. (**c**) The maximum fraction of BF filaments (plateau values of curves such as the ones shown in) as a function the product of formin FMNL2 concentration [F] and exposure duration (*T*_expo_). The solid line is an exponential fit corresponding to equation (3). Inset: *k*_obs_=*k*′_−C_+*k*′_−F_ is independent of the experimental condition. Horizontal line represents the average (Error bars: s.e.m.).

Formation of the ternary BFC complex with the FMNL2 formin was tested using set-up #1. Fast processive growth of FMNL2-bound barbed ends was rapidly arrested on exposure to CP. However, no fast elongation was seen to resume when CP was removed from the flow, possibly due to low affinity of FMNL2 for the barbed ends causing very fast dissociation of FMNL2 on CP binding. We therefore tested the formation of the ternary BCF complex starting from capped BC filaments. Filaments growing from spectrin–actin seeds were capped by CP, and subsequently exposed to varying concentration of FMNL2 for different exposure times. The rate constant for formin FMNL2 association to a capped barbed end (*k*′_+FMNL2_) was calculated as described for formin mDia1 in Fig. 3c,d. A typical kymograph of a CP-capped paused filament switching to formin-based fast elongation on exposure to 250 nM FMNL2 is illustrated in Fig. 4a. As observed with mDia1, the capped filament, following 30 s exposure to FMNL2, started fast processive elongation only after being exposed to PA for 45 s. This result indicates that at the end of the 30 s incubation of the capped filament with FMNL2, the barbed end was in the BCF and not in the BF state. FMNL2 behaves like mDia1 in Fig. 3b. Thus the data rule out a simple competition scheme between CP and FMNL2. Expectedly, the number of BC filaments that switch to BF filaments changes as the formin concentration is varied at constant exposure time (Fig. 4b), or as both are varied (Supplementary Fig. 10 and Fig. 4c). The derived association rate constant of FMNL2 to BC filaments is *k*′_+FMNL2_=0.115 μM^−1^·s^−1^, 13 times lower than free barbed ends (*k*_+FMNL2_=1.54 μM^−1^·s^−1^, Supplementary Fig. 11). As expected, the value of *k*_obs_=*k*′_−C_+*k*′_−FMNL2_ was found to be independent of the experimental conditions (*k*_obs_=0.01407±0.00153, s^−1^). Using equations (1, 2, 3) (see Methods section) we calculate *k*′_−C_=4.7±0.51 × 10^−3^ s^−1^and *k*′_−FMNL2_=9.4±1.0 × 10^−3^ s^−1^ (*N*_BF_/*B*_0_=0.334±0.026). Note that CP dissociates at very similar rate from BCF state for both mDia1 and FMNL2.

In summary, FMNL2, like mDia1, binds a CP-bound barbed end and displaces CP from the barbed end. However, a much higher concentration of FMNL2 than mDia1 is required to uncap, possibly due to the lower affinity of FMNL2 for the barbed end. Consistently, pyrene polymerization assays show modest uncapping activity of FMNL2 (Supplementary Fig. 12). In conclusion, formation of a ternary BFC complex is not restricted to formin mDia1.

### Potential structural organization of the BFC complex

**Figure 5:**
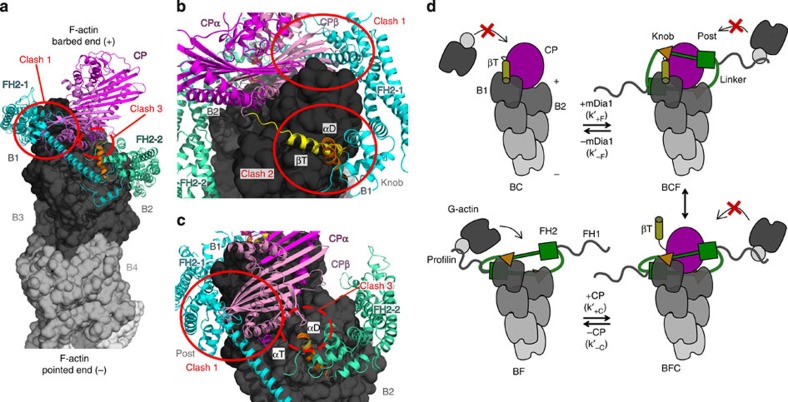
Structural clashes between CP and formin at the barbed end must cause partial dissociation of each protein in the BFC state. (**a**) Steric clashes between CP and mDia1-FH2 in the BFC complex. The 167°-twisted F-actin barbed end is depicted as surface representation (4A7N), while the α/β heterodimeric CP^30^ and the dimeric mDia1-FH2 domain (3O4X) are illustrated in ribbon diagrams. The FH2 domain hemidimers (FH2_1_ and FH2_2_) are shown in the previously defined ‘open' state^4^,^32^ and bind with an amphipathic α-helix (αD (ref. 32), orange, knob region) to the hydrophobic target-binding cleft (TBC) of actin. CP interacts with its amphipathic β-tentacle (βT, yellow) with B1, while the basic α-tentacle (αT) binds to B1 and B2. (**b**,**c**) Zoom-in of the clashes. The α-tentacle of CP is able to interact with B1 and B2 of F-actin, while there is a steric clash between CPβ and the post region of FH2_1_ (clash 1). FH2_1_ competes with CP for binding to B1-TBC (clash 2). Since CPβ and αD of FH2_2_ are in close proximity, there might be a minor steric hindrance for simultaneous binding to actin B2 (clash 3). (**d**) Cartoon depicting complex formation and dissociation of BFC, based on the present work and the structural model presented in Supplementary Fig. 13. Left panel: association of PA to the barbed end is prohibited in BC state and permitted in ‘open' BF state. Right panel: formin binds to BC by association of FH2_2_ to B1 and B2 (top) followed by displacement of β-tentacle by FH2_1_ (bottom). Similarly CP can associate with BF without inserting the β-tentacle in B1-TBC (bottom). For the detailed model, see Supplementary Fig. 13.

We next examined whether the present data and known relevant structures of CP and the formin FH2 domain dimer^29^,^30^,^31^,^32^,^33^ allow simultaneous binding of CP and formin in the BFC state, consistent with its functional properties (see Methods section). The CP αβ heterodimer binds the two terminal actin subunits (B1 and B2) of actin filament barbed ends in the helical, 167°-twisted configuration of F-actin^30^,^31^. Recent EM data at 4 Å resolution confirm this structural organization in the CapZ-capped dynactin filament^34^. In contrast, nonpolymerizable actin subunits are oriented in the FH2-actin crystal structure in a helical 180° pseudo F-actin configuration^32^, a conformation that presumably prevents the formation of main CP barbed-end contacts. Molecular dynamics studies provided insights into the BF complex in a 167°-twisted filament^35^. To challenge the simultaneous interaction of formin and CP with the standard helical filament barbed end, the mDia1-FH2 dimer (chains FH2_1_ and FH2_2_) was superimposed in the ‘open' state on the two terminal barbed-end protomers. This ‘open' state has been proposed to be adopted during G-actin association^4^. Most of the FH2-actin contacts are conserved in the 167°-F-actin context, in particular those made by the FH2 knob region, a main actin-binding element. When this structure is superimposed on the BC complex structure, steric clashes appear between CPβ and the post region of the FH2_1_ protomer. In addition, while a strong steric conflict takes place between the β-tentacle of CP and the knob region of FH2_1_, the main electrostatic interactions of CPα with B1 and B2 are not impaired (Fig. 5a–c).

We tentatively propose the following structural rearrangements to resolve the above mentioned steric conflicts. In the ternary BFC complex, the hydrophobic cleft of the terminal subunit B1 can be occupied by the knob of the FH2_1_ protomer, while the CP's β-tentacle remains unbound (Supplementary Fig. 13). This proposal is based on the low affinity of CP found here for BF, which is consistent with the reported 300-fold weakening of CP binding on removal of the β-tentacle. In contrast, the removal of the α-tentacle, abolishes binding^13^,^31^. In this putative BFC state, the strong bonds formed by αβ-CP with B1 and B2 might maintain the capping activity of CP and promote the partial dissociation of FH2_1_ from B1, thereby weakening the binding of the FH2 without affecting the contacts between FH2_2_ and F-actin (Supplementary Fig. 13). The flexibility of mDia1 due to its long linker region, may as well enable binding of formin to a CP-capped barbed end. Mutual weakening of the interface of each protein with F-actin might facilitate dissociation of BFC into BF and BC (Fig. 5d).

The destabilization of formin–actin interactions by CP should also facilitate force-induced dissociation of formin from BFC. The exposure of the post region of FH2_1_ may allow some interaction with G-actin, facilitating the displacement of CP from BFC. The displacement of formin from barbed ends by CP can also be accounted for within this model, since the strong electrostatic interactions between CP and free barbed ends^30^ are unaffected by prior formin binding. The model is schematically summarized in Fig. 5d.

## Discussion

We show that in contrast with most conventional views, CP and formin (both mDia1 and FMNL2) can simultaneously bind filament-barbed ends, albeit with mutually lowered affinities. As a result, they exhibit higher dissociation rates, which is key to the rapid displacement of one regulator by the other. Single-filament kinetics and single-molecule fluorescence explain the rapid arrest of propulsion of formin-coated beads and detachment of formin-induced bundles^3^, and provide the kinetic characterization of the molecular mechanism for CP and formin interaction dynamics with the barbed end in the ternary BFC complex.

At the current resolution of optical microscopy, it is not possible to ascertain the exact position and mechanism of interaction of the two molecules at the barbed face of F-actin terminal subunits. Hence the insight provided by biochemical function, which reflects defined protein–protein interactions, is crucial and precedes a structural study at atomic resolution.

We find that on binding to formin-bound barbed ends, CP retains its capping activity in the BFC state while its dissociation from barbed ends, monitored by resumed processive elongation by formin, is enhanced by the presence of formin. We do not directly monitor the dissociation of CP from BFC. However, the fact that the system is well-described by a first-order process of conversion of the ternary complex into only two states, BF and BC, indicates that dissociation of CP from BFC is kinetically coupled to the formation of active BF. The rates of formin and CP dissociation from BFC/BCF are roughly independent of their order of addition to barbed ends, as well as the values of the thermodynamic parameters *K*′_C_ and *K*′_F_. The derived isoenergetic square is reasonably balanced. The functional biochemical evidences strongly suggest that in the ternary BFC/BCF state, both CP and formin maintain a fraction of their original contacts with the two terminal actin subunits.

Notably, the dynamics of CP and formin have been mainly monitored in the presence of PA (except for *k*′_+F_) but may differ in the absence of actin and with profilin only. More extensive investigations are required to establish the full array of rate constants for interaction of formin itself and its interplay with CP in regimes of growth (ATP-bound terminal subunits) and depolymerization (ADP-bound terminal subunits). In spite of its lower affinity for barbed ends (even lower for CP-bound barbed ends), FMNL2 also forms a ternary complex with CP. Since the FH2 domain is conserved among diaphanous-related formins (Supplementary Fig. 14), the ability of formins and CP to simultaneously bind barbed ends may have general significance.

The displacement of formin from the barbed end by CP may have implications in the regulation of filament length. Filament bundles in filopodia are initiated by a cluster of mDia2 formins^36^,^37^, which *in vitro* rapidly assemble tens of microns long filaments before detaching. Nevertheless, filopodia are only a few micrometres long, which indicates that formin activity is regulated by other proteins. The recent evidence for CP being responsible for the tapered morphology of filopodia tips^8^,^38^ brings support to the view that CP causes formin detachment, as follows.

If we consider a typical bundle of a few tens of filaments, and assume a typical CP concentration of the order of 100 nM, we find that the population of elongating filaments steadily decreases down to few filaments in few minutes. Over that time, the bundle (that is, in a filopodium) growing at a typical rate of 100 nm·s^−1^ (ref. 39) can reach a length of a few microns (see Supplementary Fig. 15 and its caption for details). Therefore, the measured rate at which a formin-bound barbed end is capped is compatible with what can be observed in filopodia *in vivo*^22^,^39^.

Is the ability of formin to uncap CP from filament barbed ends physiologically relevant? Uncappers such as CARMIL and proteins of the CapZIP family (CIN85/CD2AP, CKIP-1 and the WASH-associated FAM21 protein) mediate rapid CP dissociation from the barbed ends, often in a site-directed fashion, suggestive of their ability to capture CP-capped filaments^40^,^41^. Uncapping of CP is mediated by multimeric WH2 proteins such as VopF (ref. 42) and Ena/VASP, also involved in filopodia formation^22^. As FH2 and WH2 domains share binding sites on the barbed face of actin, formin and WH2 proteins might use similar mechanisms to displace CP.

CP is the major barbed-end capper in filopodia of migrating cells^8^. Eps8, another capper^43^ is also found in axonal filopodia of hippocampal neurons^44^ and uses the same structural element as WH2 domains, formins, CapG, gelsolin and CP in its actin-binding mode^45^. Our results suggest that Eps8 and other proteins may rapidly displace formins from the barbed ends and vice versa.

In conclusion, CP plays a dual function in motile processes. CP helps maintaining a large pool of polymerizable monomeric actin^46^, which is essential for rapid site-directed actin polymerization, thus facilitating protrusive activities. Consistently, CP-depleted cells display short filopodia^8^. Here we propose a novel role for CP in regulating formin-based processes by limiting the dwell time of formin at barbed ends. Conversely, formin can bind a capped barbed end and restore its fast growth by displacing CP. The kinetic information provided here set the stage for *in vivo* investigations of functional interactions of CP and formin in organelles like filopodia.

## Methods

### Proteins

Actin was purified from rabbit muscle and pyrenyl- or Alexa488 succinimidyl ester-labelled (on lysines) using standard procedures. Profilin, CP, spectrin–actin seeds and the FH1–FH2–DAD domain of human formin mDia1 were expressed and purified^3^. Spectrin–actin seeds were biotinylated^25^. SNAP–FH1–FH2–DAD–His plasmid of mDia1 (ref. 47) (kindly provided by David Kovar, University of Chicago, IL), was bacterially expressed in *Escherichia coli* cells (BL21) and purified through HisTrap and gel filtration columns. Biotinylation or Alexa488 labelling of formin was done by reacting benzylguanine–biotin or benzylguanine–Alexa488 with SNAP-mDia1(FH1–FH2–DAD) following standard vendor procedure (NewEngland Biolabs).

The C-terminal FMNL2 construct carrying the terminal eight consecutive proline residues in the FH1 domain, amplified from the FMNL2 cDNA (NP_443137.2) by PCR, was cloned into *Sal*I and *Not*I sites of pGEX-6P-3 containing a N-terminal GST tag. GST–FH1(8P)–FH2–C plasmid of FMNL2 (ref. 28) (kindly provided by Jan Faix) was expressed in *E. coli* Rosetta (DE3) and purified through GST Trap and size-exclusion (Superdex 200 16/60) chromatography.

The CIN85 construct, amplified from the Cin85 cDNA (NP_114098.1) by PCR, was cloned in a modified pGEX-6P1 expression vector containing a C-terminal Strep-tag II and an N-terminal histidine–thioredoxin fusion tag. The protein was expressed in *E. coli* Rosetta (DE3) cells and purified to 98% purity through HisTrap chromatography, precision cleavage of the his–thioredoxin tag, StrepTrap and size-exclusion (Superdex 200 16/60) chromatography. The uncapping activity of CIN85 was monitored spectrofluorimetrically by measuring the restoration of barbed-end growth of CP-capped filaments from G-actin (2 μM, 2% pyrenyl labelled) in a range of CIN85 concentrations. The CIN85 concentration used in the assays provided about 70–80% uncapping.

### Bead assay for testing CP binding to formin mDia1

Biotin-functionalized polystyrene beads (Polysciences Inc.) were washed in G-buffer (5 mM Tris-HCl pH 7.8, 0.2 mM ATP, 0.1 mM CaCl_2_ and 1 mM DTT), incubated with 0.5 mg ml^−1^ neutravidin for 15 min, washed twice with 50 × excess volume G-buffer and incubated with 500 nM biotinylated mDia1 formin for 1 h. Following two washing steps as above, the mDia1-functionalized beads were split into two samples: one was loaded on the gel directly (Supplementary Fig. 1, lane 2) and the other was further incubated with 500 nM CP for 1 h. The beads were centrifuged. The supernatant with unbound CP was submitted to SDS–PAGE (lane 6). The beads were washed twice with G-buffer as above, and submitted to SDS–PAGE (lane 3). All incubations were done with 0.1% bovine serum albumin present to prevent non-specific binding of mDia1 and CP to the beads. All reactions were carried out at 4 °C. The gel was Coomassie Blue stained.

### Single-filament microscopy

The kinetics of individual filament assembly/disassembly was monitored using microfluidics coupled to fluorescence microscopy^24^,^25^ (ref. 23 for review). A 40-μm-high polydimethylsiloxane (PDMS) mould with three inlets was stuck onto a clean coverslip. First, the coverslip surface was functionalized and passivated using a 1:40 PLL-PEG-Biotin:PLL-PEG mixture (SuSos) at 1 mg ml^−1^ in PBS, for 30 min at room temperature. The chamber was rinsed and further incubated with 10 μg ml^−1^ neutravidin in PBS for 5 min. Filaments were then initiated either from biotinylated spectrin–actin seeds exposing their barbed ends in a distal position to PA, CP or formin, or from immobilized biotinylated SNAP-mDia1 (FH1–FH2–DAD) formin with their pointed ends in a distal position, thus allowing insertional filament assembly^25^. Actin was 10% Alexa488-labelled in assays where filament pointed ends were anchored by spectrin–actin seeds. When filaments were assembled from immobilized formin (anchored growing barbed ends), filaments were initiated with 15% Alexa488-labelled actin, then elongated from unlabelled actin and their dynamics were monitored^25^. All experiments were carried out at room temperature in F-buffer (5 mM Tris-Cl^−^ pH 7.8, 0.2 mM ATP, 1 mM MgCl_2_, 0.2 mM EGTA, 50 mM KCl, 10 mM DTT and 1 mM DABCO). Standard polymerization conditions are 1 μM actin and 4 μM profilin.

### Microscopy data acquisition and analysis

Actin filaments were imaged on three different microscopes. Most experiments were performed on an Olympus IX71 microscope equipped with a × 60 objective and a Cascade II EMCCD camera (Photometrics); Anchored formin renucleation activity after CP exposure (Supplementary Fig. 7) were performed on a Nikon TE-2000U microscope equipped with a × 60 objective and a sCMOS OrcaFlash2.8 camera (Hamamatsu) controlled by μManager; Experiments for the determination of the *k*′_+F_ for mDia1 (Fig. 3c,d) were performed on an ELYRA microscope (Zeiss) with a × 100 objective (numerical aperture 1.46) and an iXon 897 (Andor) camera from the ImagoSeine imaging platform (Institut Jacques Monod, Paris). Experiments were conducted in epifluorescence (mercury lamp, typical exposure duration: 500 ms) or in TIRF (typical exposure: 100 ms) at a maximal frame rate of three images per second. Images were analysed using the ImageJ Kymograph plugin. One actin subunit contributes to 2.7 nm of the filament length. Background fluorescence was subtracted automatically using the built-in rolling-ball background subtraction algorithm (rolling-ball radius 20 pixels). Typically at least 80–100 filaments were monitored in a field of view and 100% of these filaments were counted in the cumulative distribution functions (CDFs) showing the time course of the various complexes.

The fraction of filaments showing a change in growth behaviour (arrest of growth, switch from slow to fast processive growth and so on) on addition of formin or CP or PA to the flow were plotted versus time to generate cumulative distributions. The kinetics of disappearance of BFC (or BCF) linked to formation of BF and BC were analysed using the simple kinetic scheme (Fig. 2)


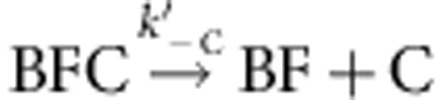



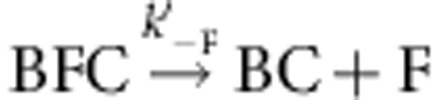


The system is described by the following differential equations


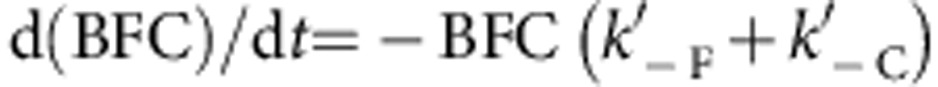



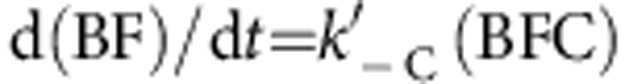



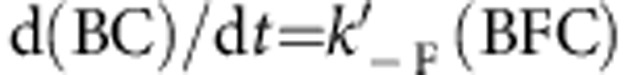


BFC, BF and BC all vary exponentially with rate constant (*k*′_−F_+*k*′_−C_). The value of *k*′_−F_ and *k*′_−C_ was derived from the relative fraction of filaments in BF and BC states as follows:


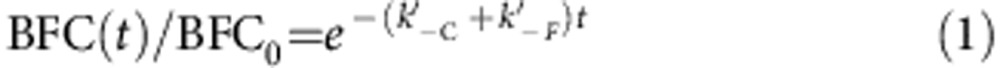



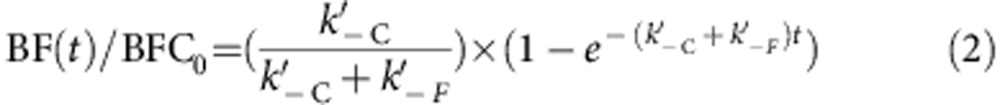



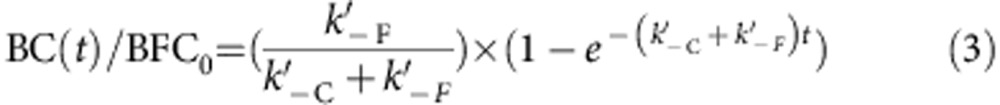


Hence *N*_BF_/*N*_BC_=*k*′_−C_/*k*′_−F_. All experiments were performed at least four times with various preparations of actin, CP, profilin and formin. The values of rate parameters given in Table 1 are representative of several measured values.

### On-rates and off-rates of CP and formin on free barbed ends

The association rate constant for formin, *k*_+F_ was measured by exposing the filaments growing in PA flow to a flow containing varying concentrations of formin along with PA. CDFs calculated for filaments that switched from slow elongation to fast elongation (
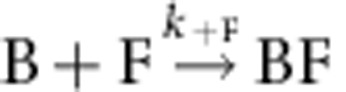
), were used to measure the on-rate of formin on the barbed end (*k*_+F_) as follows. The CDFs were analysed in terms of single pseudo exponentials (*k*_obs_) at various concentrations of formin. The association rate constant *k*_+F_ was derived as the slope of the linear increase in *k*_obs_ with formin concentration. To measure the off-rate of formin, fast-elongating filaments (BF) were observed in presence of PA until they switched to slow elongation (B). The CDF for these filaments (
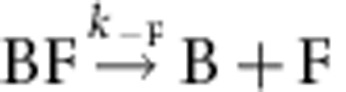
) was fitted with an exponential function to obtain *k*_−F_ (Supplementary Fig. 4). Similarly, the association rate constant of CP for barbed ends, *k*_+C_, was derived from analysis of the CDF of filaments going from slow elongation (B) to complete pause in the presence of PA and at various concentrations of CP (
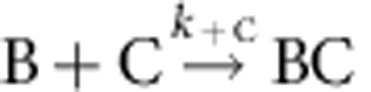
). *k*_−C_ was measured from the CDF of paused filaments (BC) switching to slow elongation in the presence of PA and absence of CP in the flow (
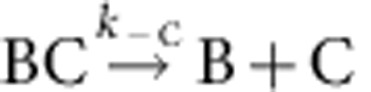
; Supplementary Fig. 5).

### mDia1 does not aggregate following fluorescent labelling

SNAP-mDia1-Alexa488 molecules, non-specifically attached on the coverslip, were exposed continuously to the acquisition laser and TIRF images were acquired at fast frame rate (three images per second). The bleaching experiments were conducted in buffer without DABCO^47^. A circle of 1 μm diameter was drawn around each spot and the integrated intensity of this area was measured using ImageJ. The average local background of all spots was measured and subtracted from each integrated intensity measurement. Majority of the spots showed either a single- or double-step bleaching profile, consistent with the dimeric nature of mDia1 (Supplementary Fig. 2).

### mDia1 remains anchored and active after exposure to CP

Presence and activity of biotin-SNAP-mDia1 specifically anchored to PEG passivated glass coverslip in microfluidics (set-up #2) are probed by exposing them alternatively to a nucleation condition (F-buffer at 25 mM KCl, 2 μM 20% Alexa488 actin and 0.4 μM profilin) and observation condition (F-buffer at 100 mM KCl, 1 μM unlabelled actin and 4 μM profilin) for 15 s each. One frame is acquired every 30 s. The localization and the time of newly formin-nucleated filaments are recorded. This allows us to localize individually all formins spots on the surface. In the control experiment, we measured the time needed for a randomly chosen sub-population of formins to nucleate a second filament after having detached their first nucleated filaments, to obtain the cumulative distribution of formin renucleation with time (Supplementary Fig. 7). In a second experiment, formins that elongate filaments (BF) are exposed to 400 nM CP to get BFC filaments and a randomly chosen sub-population of the formins that released their filaments after CP exposure (that is, BFC→BC+F) are tracked and subjected to a renucleation experiment similar to the control case described above. This leads to the cumulative distribution of formin renucleation with time after CP exposure (Supplementary Fig. 7 and Supplementary Movie 2).

### Bulk solution measurements

Barbed-end growth was monitored using pyrenyl-labelled actin fluorescence changes. A solution of 5 μM F-actin (2% pyrenyl labelled) and 5 nM CP was used as seeds. These capped filaments were diluted 25–50-fold in F-buffer containing G-actin (2% pyrenyl labelled), profilin, formin and CIN85 as indicated. Changes in pyrenyl-actin fluorescence were monitored using a Safas Xenius spectrofluorimeter.

### Structural model of CP and mDia1-FH2 bound to barbed ends

For structure display and structural modelling of the proposed BFC state, we used the PYMOL Molecular Graphics System (http://www.pymol.org) and the following available structural information regarding CP and FH2 interactions with actin: crystal structure of CP^29^, the structure of CP bound to barbed ends from cryo-EM^30^, under consideration of molecular dynamics studies^31^, the F-actin cryo-EM structure (PDB: 4A7N), the crystal structure of mDia1-FH2 domain (PDB: 3O4X) and of TMR-labelled G-actin bound to Bni1-FH2 (ref. 32) taking into account recent molecular dynamics analysis^48^. Structural information of the following studies was further considered: crystal structures of the FH2 domain of mDia1 (ref. 49), F-actin cryo-EM structure^50^ and of TMR-G-actin bound to FMNL3-FH2 (ref. 51).

The FH2 domain of the yeast formin Bni1 builds a doughnut-shaped head-to-tail dimer and it has earlier been crystallized as a continuous chain around a 180° twisted pseudo filament^32^. The main contacts between CPαβ and actin at the interface of the B1 and B2 actin protomers are not (fully) established in this pseudo F-actin configuration. In the model proposed here for the ternary BFC complex, the actin coordinates of one Bni1-FH2/G-actin heterodimer (PDB: 1Y64) were superimposed in the previously defined ‘open' state on the backbone Cα atoms of the terminal actin subunits B1 and B2, respectively, of the helical, 167°-twisted F-actin filament (PDB: 4A7N). Monomeric mDia1-FH2 domains including the C terminal, elongated αT-helix and the DAD domain (PDB:3O4X) were superimposed on the backbone Cα coordinates of Bni1-FH2 to generate an mDia1-bound F-actin barbed end. Finally, the F-actin barbed end in complex with CP (PDB data kindly provided by Y. Maeda) was superimposed on the protein backbone Cα atoms of the terminal G-actin subunit B1 (PDB: 4A7N) of the formin-bound actin filament. In this Electron Microscopy (EM) structure of BC, the CPβ subunit binds with its amphipathic β-tentacle to the hydrophobic target-binding cleft between subunit 1 and 3 of the B1 actin protomer, while the basic α-tentacle of the CPα subunit forms electrostatic interactions at the interface of B1 and B2 at the barbed end. To gain a better overview, the linker and lasso regions of mDia1-FH2 were not included into the illustration of the steric clashes in Fig. 5a–c. For the BFC model depicted in Supplementary Fig. 13, the lasso regions were separately superimposed on the post subdomains and the unstructured linker regions were remodelled to reconnect both FH2 hemidimers. This BFC model does not contain atom clashes and is in agreement with polypeptide geometry.

## Additional information

**How to cite this article:** Shekhar, S. *et al.* Formin and Capping Protein together embrace the actin filament in a ménage à trois. *Nat. Commun.* 6:8730 doi: 10.1038/ncomms9730 (2015).

## Supplementary Material

Supplementary Figures Supplementary Figures 1-15

Supplementary Movie 1 Single molecule imaging of fluorescently labelled formin forming a ternary complex with CP at the barbed end. (Complementary to Figure 1c)

Supplementary Movie 2 Anchored formins can renucleate new filaments after dissociation of filaments due to CP binding.

## Figures and Tables

**Table 1:** Kinetic parameters describing association or dissociation of CP and formin mDia1 to a formin-bound or CP-capped barbed ends in comparison with association or dissociation of CP and mDia1 to free barbed ends.

	**CP Binding to:**		**Formin mDia1 binding to:**	
**State of barbed end**	**Free (B)**	**Formin bound (BF)**	**Free (B)**	**CP bound (BC)**
Association constant (μM^−1^·s^−1^)	*k*_+C_=12.8±1.1	*k*′_+C_=0.21±0.01*k*′_+C_=0.08±0.0012*	*k*_+F_=29.1±0.59	*k*′_+F_=1.6±0.5
Dissociation constant (s^−1^)	*k*_−C_=2.0±0.38 × 10^−4^	*k*′_−C_=6.2±1.1 × 10^−3^†*k*′_−C_=1.8±0.07 × 10^−3^‡*k*′_−C_=2.02±0.04 × 10^−3^*‡	*k*_−F_=8.1±0.35 × 10^−5^	*k*′_−F_=6.2±1.1 × 10^−3^†*k*′_−F_=4.2±0.05 × 10^−3^‡*k*′_−F_=6.34±0.09 × 10^−3^*‡
Equilibrium dissociation constant (nM)	*K*_C_=0.016	*K*′_C_=9–30*K*′_C_=25*	*K*_F_=0.0034	*K*′_F_=3–4*K*′_F_=4*

The values of rate constants refer to the scheme presented in Fig. 5d. Measurements were carried out at 50 mM KCl. Rate constants are obtained by direct measurement except for *k*′_+F_. All measurements were carried out with unlabelled formins and set-up #1 unless specified otherwise. All error bars are s.e.m. Please refer to the Methods section for details.

^*^Setup 2.

^†^From BCF.

^‡^From BFC.
