# Supplementary material for: Formin and capping protein together embrace the actin filament in a ménage à trois
Source: Nat Commun. 2015 Nov 13;6:8730. doi: 10.1038/ncomms9730 (PMC4660058; doi:10.1038/ncomms9730)
Supplement: Supplementary Figures — 1-15 [file ncomms9730-s1.pdf]

Supplementary Figure 1. There is no direct interaction between formin mDia1 and CP

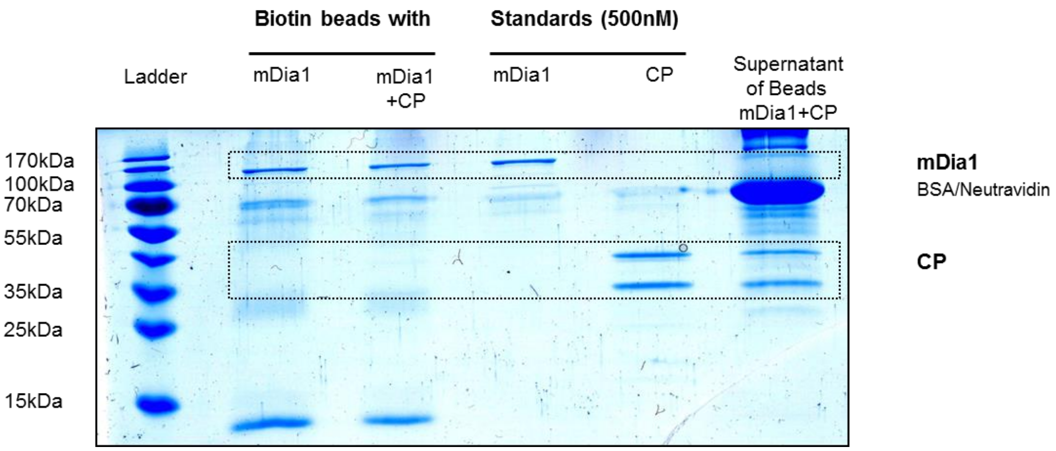

Biotinylated beads, first saturated with neutravidin were incubated either with 500 nM mDia1 or sequentially with 500 nM biotinylated mDia1 and then 500 nM CP, in a buffer containing 0.1% BSA. SDS-PAGE of pelleted beads and supernatant of mDia1 + CP sample confirm that CP does not bind mDia1 directly. 500 nM mDia1 and 500 nM CP solution standards are shown.

Supplementary Figure 2. Photobleaching tests to exclude aggregation of mDia1 formin

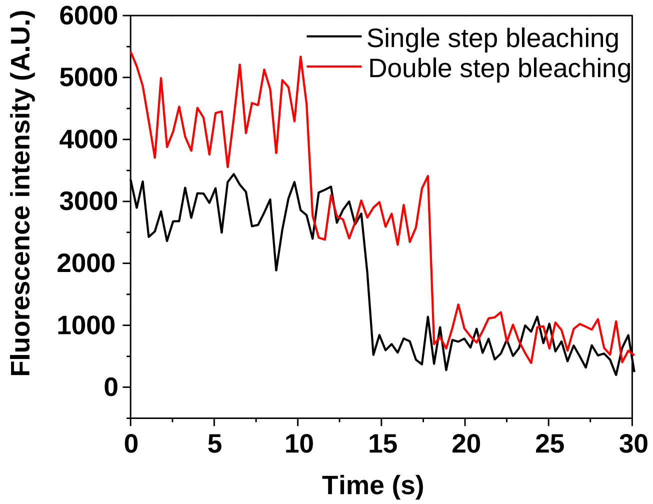

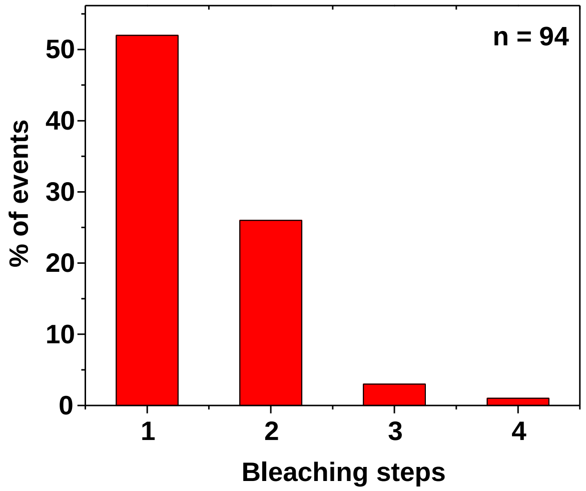

a. An example of two spots showing single-step (black curve) and double-step bleaching profile (red curve).

b. Histogram showing the number of steps leading to complete bleaching of a single spot. 94 spots were monitored. The SNAP-tagged FH1-FH2 is a dimer, thus one or two fluorophores can be bound per mDia1 molecule. Consistently, the majority of the spots (~ 80%) showed either single-step or double-step bleaching, indicating that most of the mDia1 are not aggregated. The intensity of spots with single-step bleaching behavior is in agreement with the intensity of mDia1 molecules seen in figure 1c.

Supplementary Figure 3. Histogram for capping protein associating to mDia1-bound barbed ends

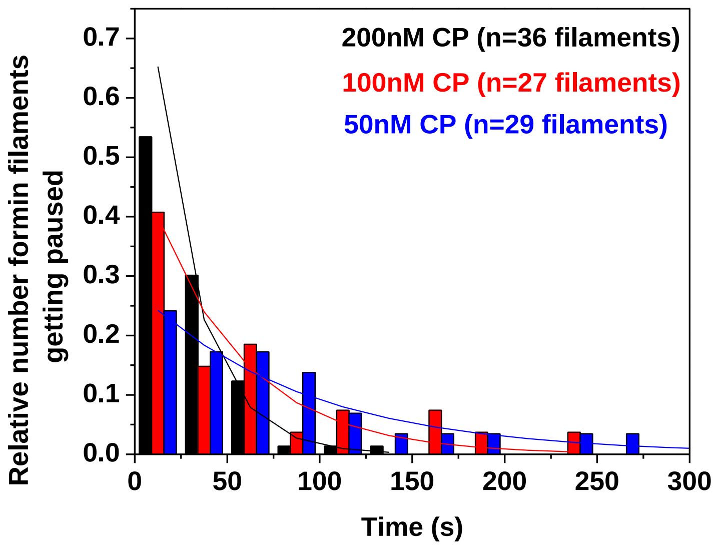

Histogram representing the time-dependent evolution of mDia1-bound filaments developing into a paused state upon exposure to 50 nM (blue), 100 nM (Red) or 200 nM (black) of Capping Protein (BF + C → BFC). The histogram has been prepared from the same data and the fit which is shown in figure 1d. Vertical bars represent the actual data of the frequency distribution and the continuous lines represent the theoretical exponential fit.

Supplementary Figure 4. Measurement of the association and dissociation rate constant of formin mDia1 to the barbed end (k_{+F}, k_{-F})

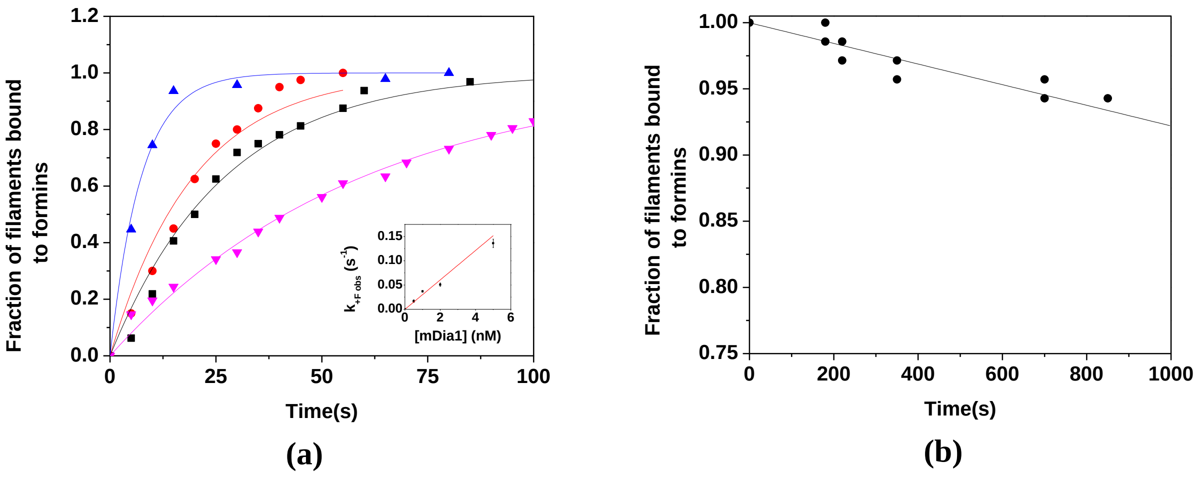

a Measurement of association rate constant for mDia1 for the barbed end. Rate of increase in fraction of barbed ends converting into an mDia1 bound barbed end (B+F→BF) in the presence of mentioned 500 pM (magenta, n=42 filaments), 1 nM (black, n=32 filaments), 2 nM (red, n=40 filaments) or 5 nM (blue, n=47 filaments) mDia1. Inset: Linear fit of the relationship between observed on-rate of mDia1 on the concentration of mDia1 gives k_{+F} = 29.1±0.59 μM^{-1}.s^{-1}.

b Measurement of dissociation rate constant for mDia1 from the barbed end. Fraction of mDia1 formin bound filaments with time (n=70 filaments), in the presence of 1μM actin, 4μM profilin. Closed circles represent actual data and the line represents the exponential fit indicating a dissociation rate of k_{-F} = 8.1±0.35 x 10^{-5} s^{-1}.

Supplementary Figure 5. Measurement of the association and dissociation rate constant of formin CP to the barbed end (k_{+C}, k_{-C})

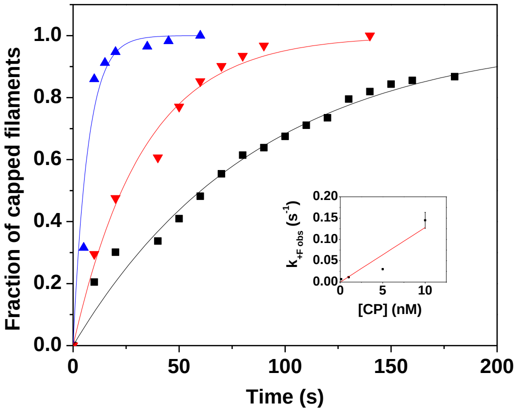

(a)

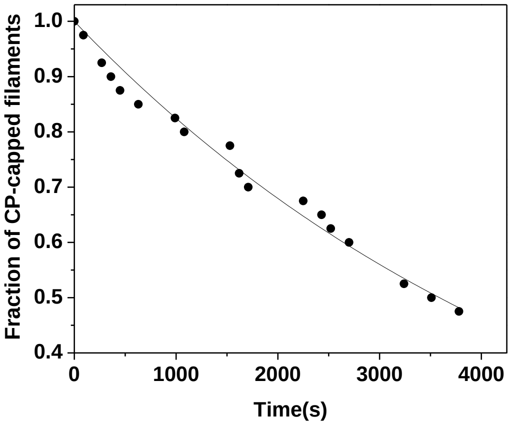

(b)

a Measurement of association rate constant for CP for the barbed end. Rate of increase in fraction of barbed ends being capped by Capping Protein (B + C → BC) in the presence of 10 nM (blue, n=31 filaments), 5 nM (red, n=61 filaments) and 1 nM (black, n=83 filaments) CP. Inset: Linear fit of the relationship between observed on-rate of CP on the concentration of CP gives k_{+C} = 12.8± 1.1 $\mu M^{-1}.s^{-1}$

b Measurement of dissociation rate constant for CP from the barbed end. Fraction of CP-bound filaments with time (n=40 filaments), in the presence of 1μM actin, 4μM profilin. Closed circles represent actual data and the line represents the exponential fit indicating a dissociation rate of k_{-C} = 2.0 ±0.38 x10^{-4} s^{-1}.

Supplementary Figure 6. BFC formation is independent of profilin-actin concentration

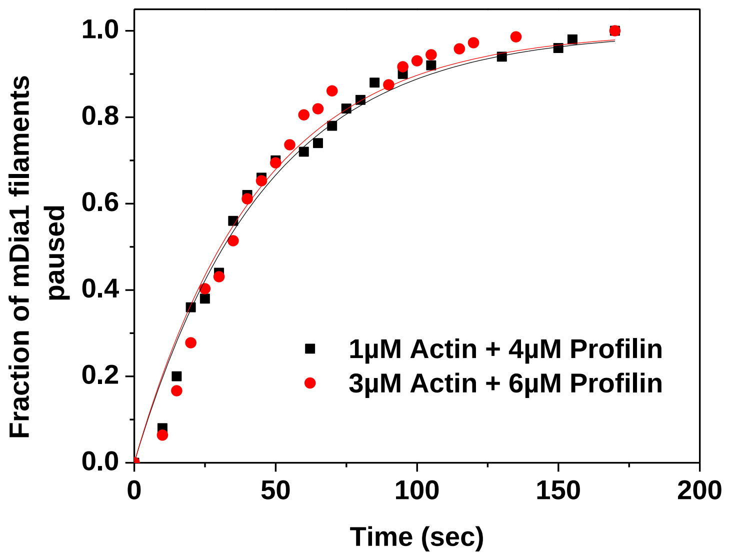

Cumulative distributions of CP association to mDia1-bound filament barbed ends at two concentrations of PA. Closed circles represent actual data and lines represent exponential fits.

Supplementary Figure 7. Surface anchored mDia1 proteins are still active following exposure to CP and release of CP-capped (BC) filaments

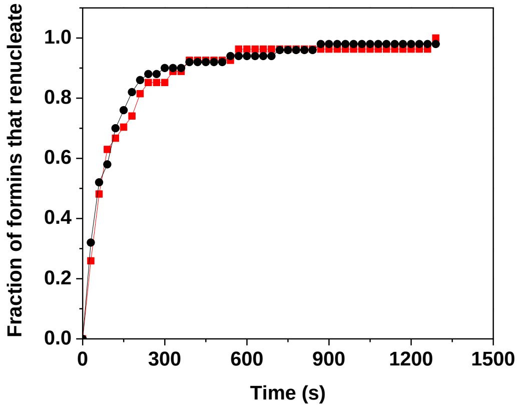

(a)

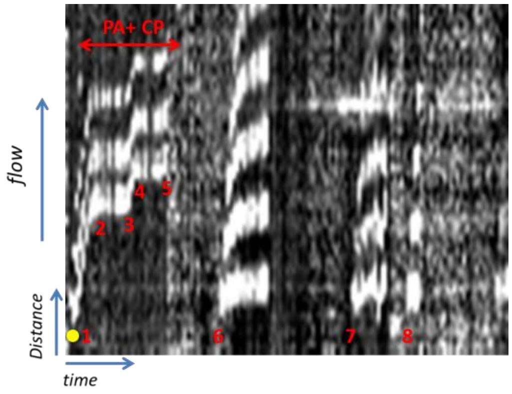

(b)

a Fraction of anchored mDia1 (setup #2) that have renucleated a filament as a function of time for a population of specifically anchored biotin-SNAP-mDia1 formins that have released a previously nucleated filament spontaneously (Red, n=27 filaments) or due to presence of 400 nM CP and the formation of the BFC complex (Black, n=50 filaments).

Anchored mDia1 were exposed alternatively to a nucleation solution (F-buffer at 25mM KCl, 2µM 20% Alexa488 actin, 0.4µM profilin) and an observation solution (F-buffer at 100mM KCl, 1µM unlabelled actin, 4µM profilin) for 15 seconds each. 1 frame is acquired every 30 seconds. The localization and time of newly mDia1 nucleated filaments are recorded. In the control experiment, we measured the time needed for mDia1 to nucleate a second filament after having spontaneously detached their first nucleated filaments, in order to obtain the cumulative distribution of mDia1 renucleation with time. In a second experiment, mDia1 molecules that elongate filaments (BF) are exposed to 400 nM CP to get BFC filaments and the sub-population of mDia1 release their filaments after CP exposure (i.e. BFC→BC+F) are tracked and subjected to a renucleation experiment similar to the control case described above. This leads to the cumulative distribution of mDia1 renucleation with time after CP exposure.

b Typical kymograph of a filament renucleating from a formin after detachment due to CP (See complementary Supplementary Movie 2). The yellow dot indicates the location of anchored

formin. The red bar indicates the period of exposure to profilin-actin + Capping Protein. Red numbers have been added to indicate the following events: a filament is nucleated (1) and elongates, until it gets capped during and pauses (2). It resumes elongation (3) indicating that CP fell off the barbed end (BFC->BF+C) and gets capped again (4). It finally detaches from the surface (5). Later, new filaments nucleate, elongate and detach from the same formin spot (6, 7, and (8).

Supplementary Figure 8. mDia1 does not significantly uncap in the absence of profilin-actin

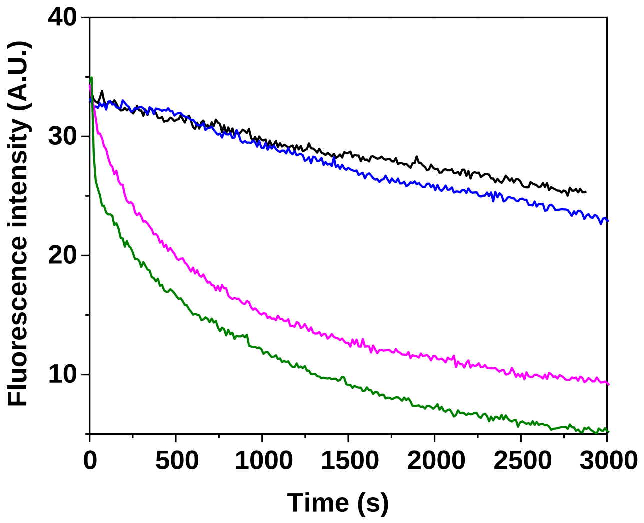

CP-capped filaments (same solution as in figure 3a) were diluted in F buffer containing 10 µM profilin, in absence (black) or in presence of either 4 µM CIN85 (magenta) or 2 nM formin mDia1 (blue) or CIN85 and formin mDia1 together (green)

Supplementary Figure 9: Measurement of formin mDia1 association rate constant to a capped barbed end (k'_{+F}) (extension of Figure 3c)

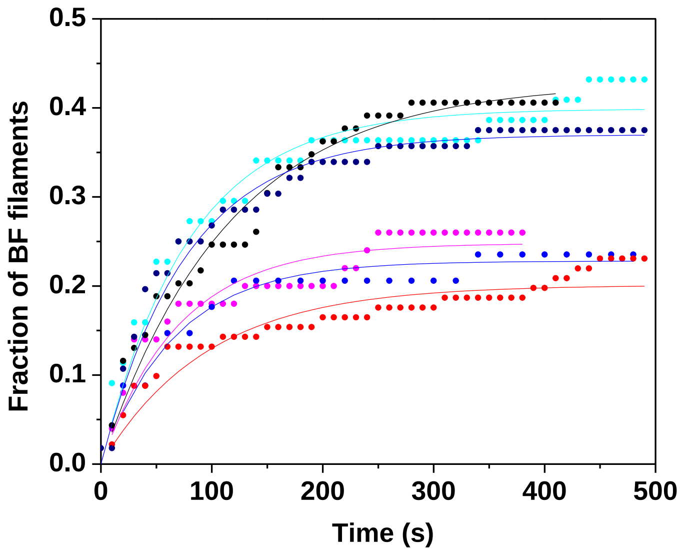

Fraction of filaments switching rapidly growing filaments (BCF → BF + C) when capped filaments were exposed to mentioned concentration of mDia1 [*F*] and time of exposure T_{expo}: red (10 nM, 30 s), blue (10 nM, 40 s), cyan (10 nM, 80 s), dark blue (15 nM, 40 s), magenta (20 nM, 30 s), black (40 nM, 30 s). Experimental data is shown by symbols and thick lines are the exponential fits respectively. Cyan, blue and red are the same curves as shown in figure 3c. Rate calculation is described in the text and shown in figure 3d.

Supplementary Figure 10: Measurement of formin FMNL2 association rate constant to a capped barbed end (k'_{+FMNL2}) (extension of Figure 4b)

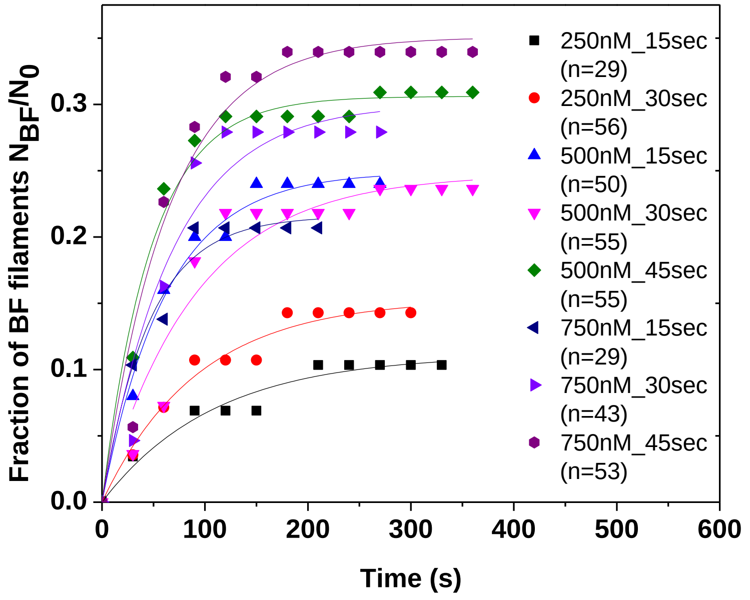

Fraction of CP-capped filaments turning to fast growing BF filaments (symbols) (BCF→ BF +C) when exposed to mentioned concentration of formin FMNL2 *[FMNL2]* and time of exposure. The respective exponential fits have also been presented (lines). The curves for 250 nM, 500 nM and 750 nM FMNL2 (30 s exposure) have also been presented in figure 4b.

Supplementary Figure 11. Measurement of FMNL2 association rate constant to a free barbed end (k_{+FMNL2})

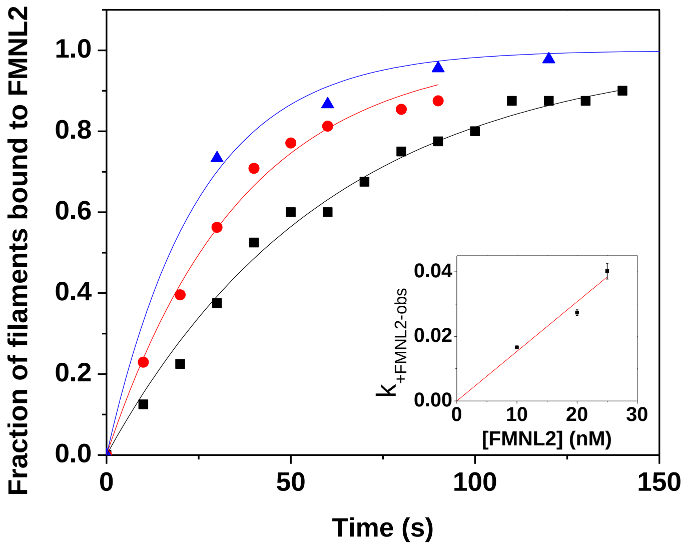

Rate of increase in fraction of barbed ends converting into a FMNL2 bound barbed end in the presence of 10 nM (black, n=40 filaments), 20 nM(red, n=48 filaments) and 25 nM (blue, n=45 filaments) FMNL2. Inset: Linear dependence of the observed on-rate of FMNL2 on the concentration of FMNL2.

Supplementary Figure 12. Formin FMNL2 binds to CP-capped filaments and uncaps via a transient BCF state

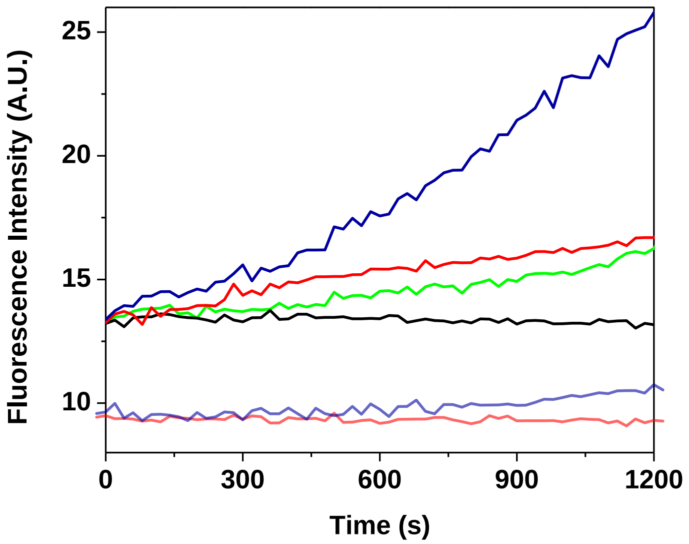

Pyrene actin polymerization assay demonstrates uncapping of capped barbed ends (BC), by formin FMNL2. Capped filaments (5 µM 2% pyrenyl-labeled F-actin, 5 nM CP) were diluted 50-fold in F-buffer containing 2 µM G-actin (2% pyrenyl-labeled) and 6 µM profilin and the following additions: none (black), 100 nM FMNL2 (red), 4 µM CIN85 (green). Light red curve is the control (2 µM actin, 6 µM profilin, 100 nM FMNL2) in the absence of CP-capped filaments. Dark blue and light blue curves are the comparative curves for 3 nM mDia1 with uncapped filaments as well as the control for 3 nM mDia1. Clearly mDia1 is a better uncapper (via the ternary state) than FMNL2. Black curve is the control for CP-capped filaments with profilin-actin.

Supplementary Figure 13. Putative model of a possible BFC conformation

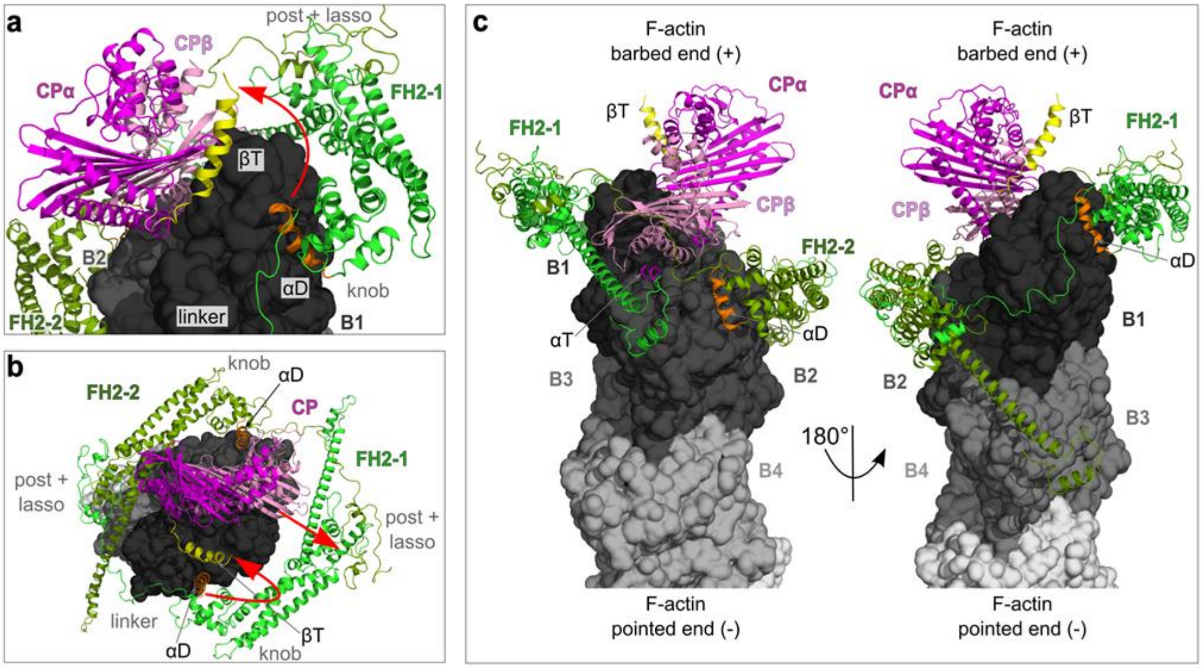

Steric clashes could be resolved by partial dissociation of CP and formin mDia1 to allow simultaneous interaction with the barbed end. As a consequence of the high affinity interaction of CPαβ with B1 and B2 the post region of $FH2_1$ might dissociate away from B1 to avoid clash 1 (**b**), while maintaining the $FH2_1$ knob contacts with B1-TBC (**a, b**). The β-tentacle probably dissociates from B1 to avoid clash 2 (**a, b**).

Supplementary Figure 14. Sequence alignment between FH2 domain of formins mDia1 and mDia2

```
mDia1-FH2        PKKVYKPEVQLRRPNWSKFVAEDLSQDCFWTKVKEDRFENNELFAKLTLAFSAQTKTSKA  60
mDia2-FH2        PKKEFKPEISMRRLNWLKIGPNEMSENCFWIKVNENKYENRDLLCKLENTFCCQEKEKRN  60

mDia1-FH2        KKDQEGGEEKKSVQKKKVKELKVLDSKTAQNLSIFLGSFRMPYQEIKNVILEVNEAVLTE  120
mDia2-FH2        TNDFD---EKK-VIKKRMKELKFLDPKIAQNLSIFLSSFRVPYEKIRTMILEVDETQLSE  116

mDia1-FH2        SMIQNLIKQMPEPEQLKMLSELKEEYDDLAESEQFGVVMGTVPRLRPRLNAILFKLQFSE  180
mDia2-FH2        SMIQNLIKHLPDEEQLKSLSQFRSDYNSLCEPEQFAVVMSNVKRLRPRLSAILFKLQFEE  176

mDia1-FH2        QVENIKPEIVSVTAACEELRKSENFSSLLELTLLVGNYMNAGSRNAGAFGFNISFLCKLR  240
mDia2-FH2        QVNNIKPDIMAVSTACEEIKKSKGFSKLLELVLLMGNYMNAGSRNAQTFGFDLSSLCKLK  236

mDia1-FH2        DTKSADQKMTLLHFLAELCENDHPEVLKFPDELAHVEKASRVSAENLQKSLDQMKKQIAD  300
mDia2-FH2        DTKSADQKTTLLHFLVDVCEEKHADILHFVDDLAHLDKASRVSVEMLEKNVKQMGRQLQQ  296

mDia1-FH2        VERDVQNFPAATDEKDKFVEKMTSFVKDAQEQYNKLRMMHSNMETLYKELGDYFVFDPKK  360
mDia2-FH2        LEKNLETFPPPEDLHDKFVIKMSSFVISANEQYEKLSTLLGSMTQLYQSIMGYYAVDMKK  356

mDia1-FH2        LSVEEFFMDLHNFRNMFLQAVKENQKRRETEEKMRRAKLAKEK  403
mDia2-FH2        VSVEEFFNDLNNFRTSFMLALKENIKKREAAEKEKRARIAKER  399
```

Identical amino acids are depicted in yellow and similar ones are colored in cyan. The two FH2 domains share 89% sequence similarity.

Supplementary Figure 15. Computation of the effect of CP-mDia1 interaction on the length of an actin bundle

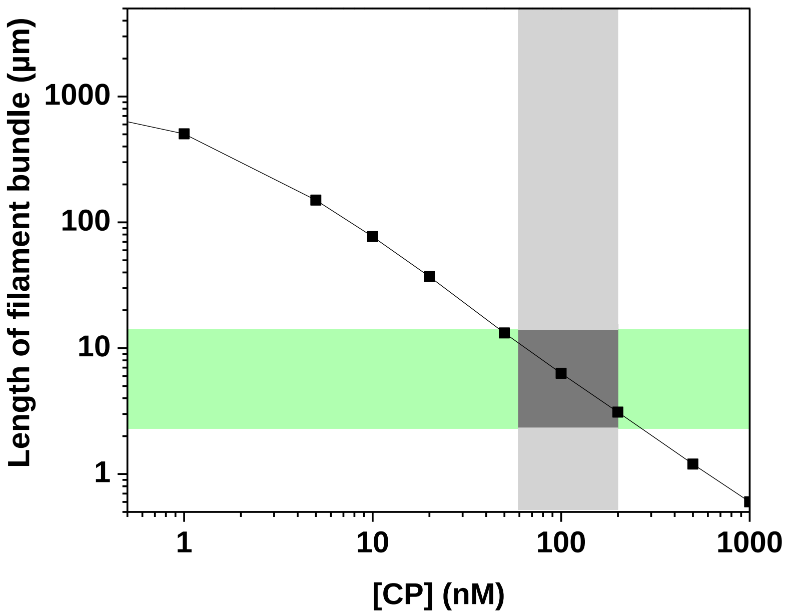

Based on previously reported numbers from *in vivo* experiments (summarized, for example in[39]) we considered a filament bundle consisting of an initial population of 30 filaments anchored to mDia1 at their barbed ends and elongating at a typical velocity of 100 nm/s[21, 39] (for e.g. in filopodia). As a result of their exposure to a concentration of Capping Protein [CP], growing filaments eventually get capped and detach from the filopodium tip, following the reaction scheme BF + C → BFC → BC (the alternate route BF + C → B + C → BC is negligible). We can numerically solve the differential equations of this reaction scheme and, using the rate constants determined in this paper (Table 1), compute the number of growing filopodium filaments (BF) versus time, or versus the length reached by the still growing filaments. When the growing filament population is down to 6 filaments, i.e. 30% of its initial population, it can no longer support filopodium elongation and stalls[39]. We find that for [CP] in the range of 50-200 nM (light gray shaded region) the filopodium reaches a length comprised between 3 and 13 µm (green shaded region).
